# The Impact of the COVID-19 Pandemic on the Orthopedic Residents: A Pan-Romanian Survey

**DOI:** 10.3390/ijerph19159176

**Published:** 2022-07-27

**Authors:** Flaviu Moldovan, Adrian Gligor, Liviu Moldovan, Tiberiu Bataga

**Affiliations:** 1 Department of Orthopedics-Traumatology, “George Emil Palade” University of Medicine, Pharmacy, Science, and Technology of Targu Mures, 38 Gh. Marinescu Street, 540142 Targu Mures, Romania; tbataga@gmail.com; 2 Biomedical Research Center, “George Emil Palade” University of Medicine, Pharmacy, Science, and Technology of Targu Mures, 1 Nicolae Iorga Street, 540088 Targu Mures, Romania; adrian.gligor@umfst.ro; 3 Faculty of Engineering and Information Technology, “George Emil Palade” University of Medicine, Pharmacy, Science, and Technology of Targu Mures, 1 Nicolae Iorga Street, 540088 Targu Mures, Romania; liviu.moldovan@umfst.ro

**Keywords:** orthopedics, COVID-19, coronavirus, pandemic, residency, stress factors

## Abstract

The COVID-19 pandemic has brought unprecedented challenges, with a potential stress which might affect the education of resident doctors in the field of orthopedics and traumatology. Its repercussion on the residents’ strain and training routes is not well known. After two years of pandemic, this paper aims to analyze the repercussion of the coronavirus disease 2019 (COVID-19) on education, medical training, and the mental well-being of Romanian resident doctors in orthopedics and traumatology. In January–February 2022, an electronic questionnaire was distributed to all orthopedic resident doctors in the 12 residential training centers in Romania. Participants (*n* = 236) were resident doctors with an employment contract and professional activity during the COVID-19 pandemic. Resident doctors who did not work during this period were excluded. An online survey generator was used to electronically create the questionnaire. Statistical analysis was performed in Matlab version R2022a, with the support of Statistics and Machine Learning Toolbox Version 12.3. Descriptive statistics were performed for the standardized questions, while for the open questions, answers were collected by topic. The results of the Chi-square test indicate that there is a statistically significant association regarding the prevalence of infection among residents involved in the treatment of patients with COVID-19 (*p* = 0.028), and the influence of secondment in COVID-19 sections (*p* = 0.0003). The infection of residents is not related to their affiliation with a particular medical training center (*p* = 0.608), gender (*p* = 0.175), the year of study in residency (*p* = 0.733), the age group (*p* = 0.178), and the secondment period (*p* = 0.114). Residents who participated in the study had an overall well-being index of 13.8 ± 5.7, which indicates a low level of well-being for a large number of residents. Residents who would like to choose a new residency specialization, or would choose a non-medical career, had reduced average WHO wellness rates, as the risk of infection is associated with the treatment of patients with COVID-19 and secondment in COVID-19 sections. The findings of this study may help residency training centers to develop robust programs that can alleviate the impact of this pandemic. Some major changes will be needed to be integrated into residency training programs around the world. Emphasis should be placed on electronic educational portfolios, simulation of surgical processes, and distance learning, all of which have a high potential for health and safety, as well as for the moral support of residents.

## 1. Introduction

Orthopedics is a complex medical specialization that brings together several types of medical activities, in which outpatient health management is combined with inpatient surgeries. Therefore, the residency program in orthopedics should stimulate the development of clinical skills, which can then be combined with surgical training to treat patients with orthopedic conditions.

Medical education and training in Romania is a sectorally regulated process under the strict supervision of the Ministry of Health, which, due to the size of the public health crisis caused by the coronavirus disease, required significant adjustments.

For about two years, the global pandemic COVID-19 has been the biggest threat faced by the Romanian health system, and the management of this crisis around the world has taken into account socio-cultural aspects in the context of an epidemic [1,2].

The COVID-19 pandemic rapidly overwhelmed the resources of the Romanian medical system, having far-reaching effects on it, and requiring changes of unprecedented proportions in the way healthcare is provided in Romania. Medical staff were redeployed to provide first-line care for patients infected with COVID-19, in medical facilities that were often converted into ad hoc intensive care units, while elective operations in orthopedics and trauma departments were suspended.

From April 2020, all elective surgeries were canceled in most hospitals in Romania, due to the need to relocate health care workers to COVID-19 wards.

From the beginning of the coronavirus pandemic, orthopedic residents, as well as other specialties, were brought to the forefront [3]. With the drastic decline in the number of elective surgeries [4] in hospitals around the world, some residents were transferred to intensive care units, hospital wards, and COVID-19 outpatient departments. The transfer did not reflect the gender distribution, which is higher in males [5,6,7].

The body of residents was divided into teams, some were given thetask of serving patients with COVID-19, other teams were given orthopedic tasks, and some of the residents werekept in quarantine [8]. The pandemic has significantly affected residents’ academic teaching, academic examinations, surgical exposure, practical training, and mental stress related to COVID-19 duties [9].

The period of preparation for residency follows a period of study in medicine, which is also a stressful time [10], and for which mitigation attempts must be made [11].

Since the pre-pandemic period, some studies have suggested that burnout levels are high among residents, and may be associated with depression and problematic patient care [12]. With the onset of the COVID-19 pandemic, the education of orthopedic residents has faced major challenges. Almost all aspects of orthopedic training have been disrupted, ranging from residency entrance exams, current training in university clinics, to specialist exams.

During the pandemic, the academic program of medical graduates, who are now enrolled as residents in years one and two, took place mostly online, with no internships in hospital. Once enrolled in hospitals, the number of operations they attended was extremely low because, in most hospitals, elective interventions were suspended. Thus, the training of residents has undergone a transformationprocess, in which it has gone from problem-based, on-site learning, to online learning which is based on virtual models [13,14]. All of these situations have had a significant impact on the training of residents.

The repercussion of the coronavirus disease on the development of residency orthopedic courses is an area of great interest in research, as the way in which medical training was conducted during this period will have immediate, as well as long-term consequences, in the field of orthopedics.

The impact of the pandemic on health workers has been studied [15], and it was found that they were affected by fear of contagion, distress, and anxiety [16]. However, its impact on the stress and subsequent careers of healthcare professionals is not yet well understood.

During the pandemic, there were medical surgical specializations that had to redistribute some of its residents to COVID-19 treatment units. Among them was orthopedics specialization, which, by reducing the number of residents in wards, and receiving mostly non-orthopedic tasks, has generated a series of questions regarding the future of training in this field. Therefore, studies are needed that focus on the residents’ views about the drastic changes in current clinical and training activities. Depending on the current pandemic situation, the opinion of the residents can contribute to the worldwide adaptation of the orthopedic residency program curriculum. For these reasons, the repercussion of the coronavirus disease on orthopedic residents, and their training, needs to be investigated in detail. Our work experience in the orthopedics-traumatology department entitles us to investigate the situation of professional training in this field, and to identify new mechanisms of surgical education.

Based on the main lines of research identified in the literature review, we have formulated the following research hypotheses:

**Hypothesis** **1** **(H1).** *Treatment of patients with COVID-19 is directly related to the infection of residents.*

**Hypothesis** **2** **(H2).** *Affiliation of residents with a particular medical training center is directly related to their infection.*

**Hypothesis** **3** **(H3).** *Gender has an impact on infection prevalence.*

**Hypothesis** **4** **(H4).** *The year of study in residency correlates to an increased risk of infection.*

**Hypothesis** **5** **(H5).** *Belonging to a certain age group is associated with an increased risk of infection.*

**Hypothesis** **6** **(H6).** *Secondment in COVID-19 sections is associated with an increased risk of infection.*

**Hypothesis** **7** **(H7).** *The period of secondment has an increased influence on the infection rate of residents.*

Given the impact on the training environment of residents, and the increased exposure to stress, the aim of this study is to highlight the experience of orthopedic residents, who work in specific orthopedic and non-orthopedic tasks. Another aim, which has not been sufficiently evaluated so far, is to highlight the circumstances of residents in orthopedics, and bring to mind their mental state while they perform risky and stressful work. It also aims to evaluate the repercussions of coronavirus disease on the professional training and well-being of Romanian residents in orthopedics and traumatology, during the two-year period affected by the pandemic.

## 2. Materials and Methods

The methodology of this study consisted of:

1. Study design and selection of study participants;
2. Development of a questionnaire;
3. Participants’ recruitment and questionnaire distribution;
4. Data collection and statistical analysis.

### 2.1. Study Design and Participants

Resident doctors, from clinical hospitals located in 12 residential training centers in Romania, were sent an electronic questionnaire. The survey was developed and written in Romanian. The study protocol was approved by the Emergency County Clinical Hospital, from Targu Mures.

### 2.2. Development of the Questionnaire

The sections of the questionnaire were developed primarily by studying the medical literature and adjustment of selected models, followed by short meetings with a small group of physicians residing at a single Romanian emergency clinical hospital.

In January 2022, we searched for publications in PubMed, EMBASE (OVID) and Web of Science using the search terms of the Boolean operator “COVID-19” and “orthopedics” and (“resident” or “internship” or “training”), in order to identify areas that could affect the training of residents in orthopedics, but also for other surgical medical specializations that could present information of interest to orthopedics.

Khusid et al. [17] consider that the training of resident doctors should be researched from the perspective of seven aspects: redistribution; physical and mental well-being; operational experience and surgical training; training and integration in telehealth; didactic learning; education for medical students and applications for residency; and academic leadership. Abbas et al. [18] studied the effects of the pandemic on medical students using a four-section questionnaire: demographics; stressors; the WHO Welfare Index; and the management of stress and resources.

In the next stage of the study we built the questionnaire, which consists of five sections: (1) *General data and repercussions felt by the pandemic*; (2) *Clinical activity* (Clinical activity on duty/emergency room/outpatient ward; Clinical activity of hospitalization/discharge; Clinical activity in the operating room); (3) *Academic training activity in specialization* (Orthopedic training; Academic activity); (4) *Mental health* (Stressors; Assessment of well-being-WHO questionnaire); (5) *Issues considered relevant by respondents*.

The first section of the questionnaire, *General data and repercussions felt by the pandemic*, consisted of questions identifying the training center where the resident works; the year of preparation for residency; age; sex; and the impact of the pandemic: whether he/she treated COVID-19 positive patients; whether he/she was seconded, and for how long, in COVID-19 wards; if he/she tested positive for COVID-19; and if quarantined.

The second section, of *Clinical activity*, was divided into three subsections that were dedicated to the study of clinical activity. This is the predominant practical component of the professional training of resident orthopedic doctors. In each of the three sections, similar response options were used to assess the difficulty of the aspect discussed during the pandemic, compared to the period before the coronavirus pandemic. The evaluation was performed using a five-step Likert scale, between the extremes: “much more difficult in a pandemic compared to an ante-pandemic” to “much easier in a pandemic compared to an ante-pandemic”.

#### 2.2.1. Clinical Activity on Duty/Emergency Room/Outpatient Department

Residents were assessed through six questions regarding difficulties in managing the flow (number) of patients, examining patients, and obtaining the opinion of a specialist/primary care physician when dealing with complicated cases. It also evaluated the way in which plaster cast immobilization services were performed; the provision of personal protective equipment when in contact with patients; and the extent to which their own activity was affected by the risk of COVID-19 infection.

#### 2.2.2. Clinical Activity of Hospitalization/Discharge

Six questions were asked regarding appropriation of the administrative aspects related to medical flows, from clinical activity with regards to the preparation of hospitalization/discharge documentation, to hospitalized patient follow-up. Also, in this section we evaluated the way in which patients were referred for laboratory and radiological investigations; the availability of appropriate medicine/equipment for medical treatments; collaboration with other departments to obtain medical information about patients; and how to cooperate with administrative staff.

#### 2.2.3. Clinical Activity in the Operating Room

The questioning continued with six questions that assessed how a patient’s preoperative preparation for surgery was performed; procurement and preparation of the implant for surgery; and the scheduling of intervals for surgery. The way in which activity in the operating room contributes to professional training, through the accumulation of practical surgical experience facilitated by the willingness of the surgeon to involve the resident, was also evaluated.

The third section, *Academic training activity in specialization*, was divided into two subsections, which were dedicated to the study of academic activity of the training specialization. This is the component with theoretical preponderance of the professional training of orthopedic resident doctors. During this period, the affective and psychomotor domains were assessed by case simulations, with standardized patients and practical clinical examinations in the hospital. Most students and residents were subjected to summative assessments, in which the cognitive domain was evaluated by grid tests. These subsections were designed in order to know the residents’ perspective on curriculum change and clinical work.

#### 2.2.4. Orthopedic Training

This control subsection of the questionnaire is also assessed on a Likert scale, with the same five values. The issues evaluated so far were checked in order to test the veracity of the answers.

#### 2.2.5. Academic Activity

In this subsection, the survey contained four questions, which assessed whether the respondent was completing his/her academic training through master’s or doctoral studies; and whether he/she carried out scientific research activities. If so, the respondent was asked three additional questions to study the availability of time for scientific research; the degree of difficulty in recruiting patients for research; and for conducting prospective/retrospective research. The involvement in the training of students was also assessed. For those involved it was evaluated with three additional questions related to: preparation for practical internships; interaction with students; and examination of students.

Affirmative answers to the last two questions were evaluated on a five-point Likert scale, similar to the one presented for evaluation in previous sections of the questionnaire.

The next section of the questionnaire, the fourth, was dedicated to *Mental health* and was composed of two evaluation subsections.

#### 2.2.6. Stress Factors

The survey included stress factors that were designed by a small group of Romanian residents. At the beginning of the section, a question was asked to assess the respondent’s stress level in the circumstances created by coronavirus disease. The next two evaluations were aimed at the repercussion of coronavirus disease on the personal option for a new residency specialization, and also for the option to choose a non-medical career. In the affirmative case of opting for a new residency specialization, the respondents were asked about the new field of residency on which they wished to focus.

#### 2.2.7. Assessment of Well-Being (WHO Questionnaire)

This subsection of the questionnaire assessed the respondent’s well-being, using the WHO welfare index [19]. The measuring instrument was composed of five conditions that residents evaluated according to their own condition. The questionnaire has a high clinical relevance, being one of the most often used tools for personal assessment of psychological condition [20]. The WHO-5 questionnaire is available in a large number of languages, including Romanian [21]. The maximum value of the score that can be obtained by applying the questionnaire is 25; values that are below 13 describe a weak condition, and an indication for depression testing [21].

The last section of the questionnaire addressed an open question that permitted residents to present personal issues that they consider relevant to how the COVID-19 pandemic affected their professional work and personal life, as well as proposals for solving them.

A research group in medical education at the “George Emil Palade” University of Medicine, Pharmacy, Science and Technology of Targu Mures, which consisted of doctors, researchers in the field of medical education, and residents, ensured the scientific relevance of the questionnaire by reviewing it.

After designing the questionnaire, it was tested on 12 respondents who met the requirements to participate in the survey. It tested the intelligibility, readability, and plenitude of the questionnaire. The results allowed improvements in content and format, with regards to how to formulate items in the clinical activity section, but also their sequence, improvement of the graphical interface to be as user-friendly as possible, and the grid for assessing well-being by using the WHO-5 questionnaire. The data collected in this test were not used in the main research.

### 2.3. Participants’ Recruitment and Questionnaire Distribution

The questionnaire was conceived and deployed with the Online Form Builder, developed by Google. The invitation for participation was submitted to the heads of orthopedics-traumatology departmentsin all of the 12 residency training centers. They were invited to analyze the survey methodology, the content of the questionnaire, and to decide whether the orthopedics department they lead would participate in the study, with a deadline of one month. Department heads that declined to participate in the study were asked to state reasons for this decision, in order to explore possible impediments to the smooth running of the study.

For those departments that chose to participate in the survey, the distribution of the questionnaire was done by sending an electronic message to eligible residents, in which the electronic address of the questionnaire was indicated. Eligible participants were resident doctors with an employment contract in the clinical hospitals located in the 12 residential training centers, and whose professional activity was affected by the COVID-19 pandemic. Resident doctors who did not work during this period were excluded from the study.

At the beginning of the questionnaire, a request for obtaining the consent to participate in the study was posted, which ensured its voluntary character. The survey was anonymous and lasted between 10 and 15 min. The questionnaire was designed to allow aquestion to be omitted if it was considered inappropriate by the respondent, and to complete a single answer option for each question.

### 2.4. Data Collection and Statistical Analysis

Between 28 January 2022 and 25 February 2022, the data completed by the residents were collected, after which their statistical processing was performed. The data were filtered, analyzed primarily, and transferred to Microsoft Excel, GNU PSPP and Matlab for further processing. In justified situations where respondents did not answer certain questions, the related data were excluded from all statistical processing.

The methodology for processing the collected data consisted of a separate analysis for each of the five sections of the questionnaire. The percentage of answers to all questions was noted. For the quantitative variables, the mean and standard deviation were calculated. Statistical analysis was performed with Statistics and Machine Learning Toolbox Version 12.3 from Matlab R2022a (The MathWorks, Inc., Natick, MA, USA).

#### 2.4.1. General Data and Repercussions Felt by the Pandemic

The categorical variables (university center where the resident works, year of preparation for residency, age, sex, involvement in treating positive patients and personal condition of coronavirus infection and quarantine) were expressed as a percentage, in relation to the total number of respondents.

*Clinical activity: on duty/emergency room/outpatient ward, inpatient/outpatient, in the operating room.*

Descriptive statistics were performed for these three sections, and quotas were employed to evaluate the difficulty of conducting clinical activity during the pandemic.

*Training in Orthopedics* and *Academic Activity* were assessed by descriptive statistics. The quotas were employed to evaluate the numberof residents who managed to improve their training through studies conducted in parallel with the current activity, at master’s and doctoral level, and for scientific research and professional training activities for students, in order to assess the degree of availability for other forms of professional development, but also the degree of difficulty for their own training in orthopedics.

*Stressors* were assessed by descriptive statistics, and quotas were employed to evaluate stress levels during the pandemic, the influence of stress on the number of residents who reconsidered their choice as a residency specialization, or even medicine as a career.

In order to test the validity of the formulated hypotheses, and to determine whether there is a statistically significant association between variables of interest, we performed the Chi-square test of independence.

#### 2.4.2. Assessment of Well-Being

We started with a descriptive statistic regarding the frequency of the well-being index, after which we calculated for all respondents the mean and the standard deviation. After that, we made a comparison of the well-being index for the two groups of participants: residents with a personal preference for a new specialization, or even a non-medical occupation; and those who wanted to continue their medical career. These data were taken from the previous section of the questionnaire, in which the stressors were assessed. It employed the Chi-square test, with corrections where necessary, in order to compare the two groups.

Confidence intervals were calculated for a 95% confidence level for each performed statistics analysis. GNU PSPP software and Matlab with Statistics Toolbox were used to determinethe statistical dependence.

For the final open question in the *Other issues that you consider relevant* section, residents presented a multitude of difficulties and challenges they encountered during the pandemic, at the hospital level, or related to the residential training system, but also some proposals for solving them. Some of them were considered relevant and were retained as potential solutions. The selection criteria of those topics retained consisted of approaching some aspects regarding the general problems of the residents in orthopedics, as well as their highlighting by more than seven residents.

## 3. Results

The orthopedic-traumatology hospital wards that agreed to participate in the study cover all 12 residency training centers. They distributed the questionnaire to their residents within an acceptable time frame. There were 244 responses, of which 8 (3.27%) were excluded because they did not give their informed consent. In the end, 236 residents were included in the study, which represents a proportion of 40.20% of the total number of 587 residents in orthopedic-traumatology existing at national level. This is a representative sample for the study group, as it is larger than the sample size of 233 respondents (95% CL, CI = 40.20 ± 3.97%, ±5% acceptable margin of error), which ensures that the study is not underpowered.

### 3.1. General Data and Repercussions Felt by the Pandemic

The characteristics of the residents who chose to participate in this study are presented in Table 1.

Most of the participants included in the study were men (*n* = 194, 82.20%), mostly between the ages of 25 and 30 (*n* = 212, 89.83%). The distribution of residents by year of residency training was uniform (year 1, 19.92%; year 2, 23.73%; year 3, 16.53%; year 4, 20.76%; grade 19.07%). Additionally, the distribution, taking into account the training center, is estimated to be proportional to the size of the training centers and relevant at national level.

Most residents treated patients who tested positive for COVID-19 (*n* = 170, 72.03%). Some of the residents were seconded to COVID-19 sections (*n* = 67, 28.39%). Also, most of the residents tested positive for COVID-19 (*n* = 141, 59.75%), and were quarantined (*n* = 141, 59.75%).

### 3.2. Clinical Activity

**Figure 1:**
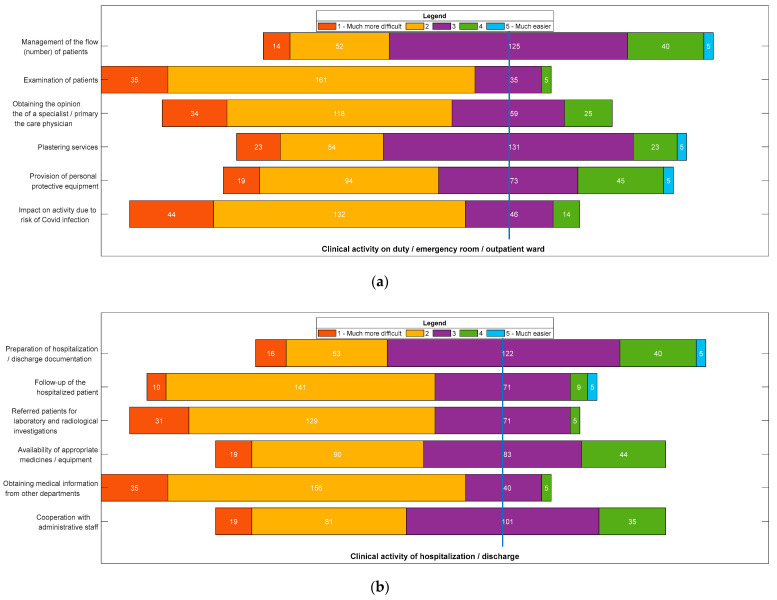

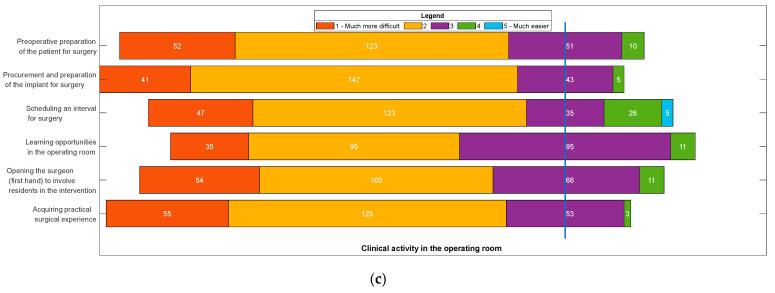
Likert scale of the degree of difficulty regarding the development of clinical activity: (**a**) Clinical activity on duty/emergency room/outpatient ward; (**b**) Clinical activity of hospitalization/discharge; (**c**) Clinical activity in the operating room. Steps on the Likert scale: (**1**) Much more difficult in pandemic than in ante-pandemic; (**2**) More difficult in pandemic compared to ante-pandemic; (**3**) In the pandemic, as well as in ante-pandemic; (**4**) Easier in pandemic than in ante-pandemic; (**5**) Much easier in the pandemic compared to the ante-pandemic period.

Figure 1 shows the degree of difficulty encountered by residents in conducting clinical work. In the clinical activity on duty/emergency room/outpatient ward, the most difficult aspect in the pandemic, compared to ante-pandemic, was the examination of patients (*n* = 196, 83.05%). In the clinical activity of hospitalization/discharge the most difficult aspect in the pandemic compared to ante-pandemic was obtaining information from other departments (*n* = 191, 80.93%). In clinical activity in the operating room, the most difficult aspect in the pandemic compared to ante-pandemic was the procurement and preparation of implants for surgery (*n* = 188, 79.66%).

### 3.3. Academic Activity and Training in Orthopedic Specialization

Table 2 shows the share of residents who managed to improve their training through studies at master’s level and doctorate, and who carried out scientific research activities and were involved in the professional training of students.

**Figure 2:**
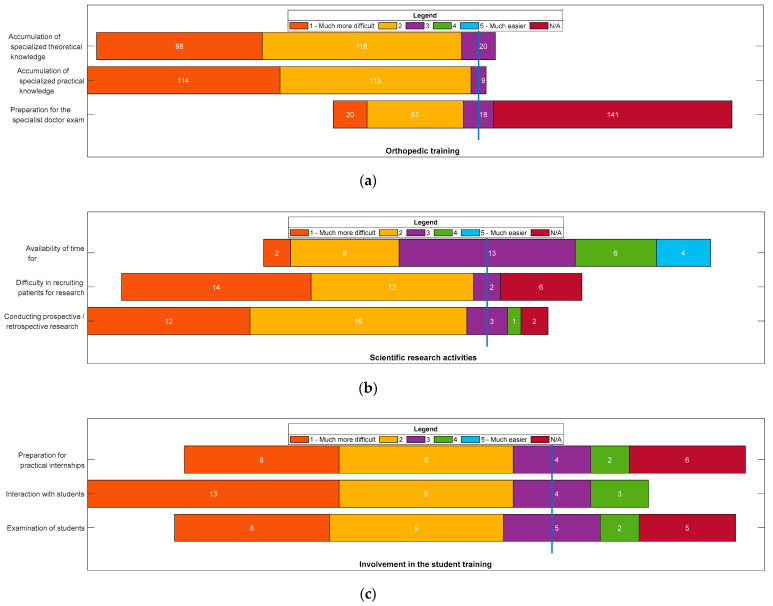
Likert scale of the degree of difficulty regarding academic training activity in specialization: (**a**) Orthopedic training; (**b**) Scientific research activities; (**c**) Involvement in the training of students. Steps on the Likert scale:(**1**) Much more difficult in pandemic than in ante-pandemic; (**2**) More difficult in pandemic compared to ante-pandemic; (**3**) In the pandemic, as well as in ante-pandemic; (**4**) Easier in pandemic than in ante-pandemic; (**5**) Much easier in the pandemic compared to the ante-pandemic period; N/A not applicable.

Figure 2 shows the degree of difficulty encountered by residents in carrying out the activity of improving their medical training in the field of orthopedics, conducting scientific research, and the professional training of students.

In the training of orthopedic specialization, the most difficult aspect in the pandemic compared to ante-pandemic, was the accumulation of specialized practical knowledge (*n* = 227, 96.18%). In carrying out scientific research activities, the most difficult aspect in the pandemic, compared to ante-pandemic, was the performance of prospective/retrospective research (*n* = 28, 82.35%). For residents involved in training students, the most difficult aspect in the pandemic, compared to ante-pandemic, was interaction with students (*n* = 22, 75.86%).

Table 3 shows the level of stress of residents during the pandemic, in which residents were affected by higher-than-usual stress levels; residents who had the level of stress about the same as usual; and residents who indicated a lower level than usual.

There were 35 residents which have a personal preference for a new residency specialization, as seen in Table 4. Only three residents would choose a non-medical career.

The internal consistency of the final applied questionnaire items was analyzed using Cronbach’s alpha test. The computed score of 0.8871 indicates a good internal consistency.

### 3.4. Correlation Assessment of Categorical Variables

**Figure 3:**
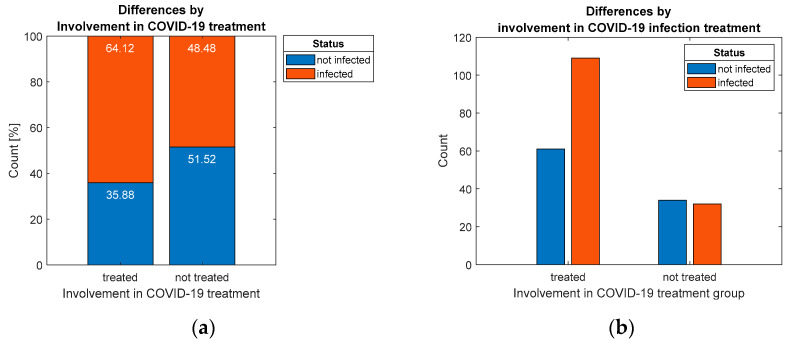
Distribution of respondents by: (**a**) percentage and (**b**) count, considering differentiation by involvement in COVID-19 infection treatment.

The results of the first test of independence Chi-square indicate that there is a statistically significant association (*p* = 0.028) regarding the prevalence of infection among residents, which was detected between the group that was involved in the treatment of patients with COVID-19, and those that were not, which is also illustrated by the proportions in Figure 3. The results of the empirical analysis show that the formulated research hypotheses H1 have been confirmed, and the treatment of patients with COVID-19 is directly related to the infection of residents.

**Figure 4:**
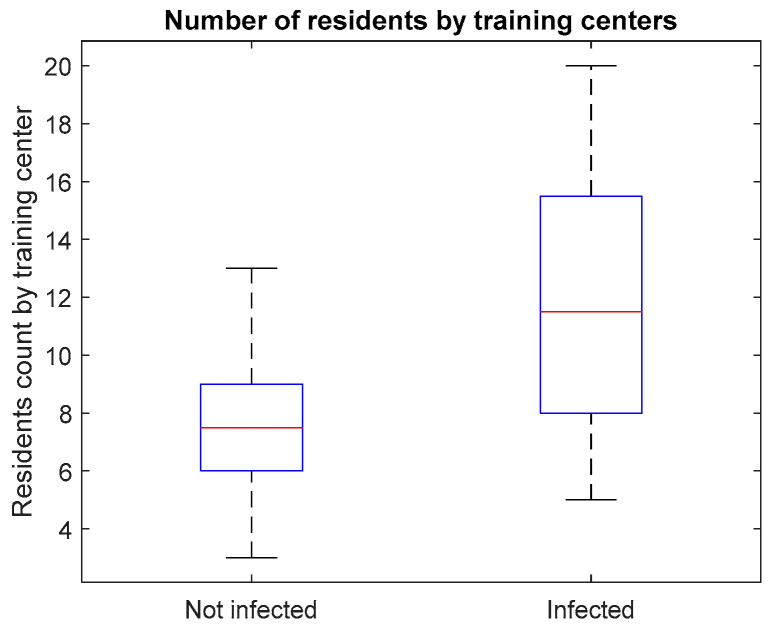
Distribution of respondents considering their count on training center.

Hypothesis H2, regarding the fact that infection of residents is directly related with their affiliation in a particular medical training center, is not confirmed by the Chi-square test of independence, which shows that no statistically significant association (*p* = 0.608) could be established. This is also illustrated by the proportions in Figure 4.

**Figure 5:**
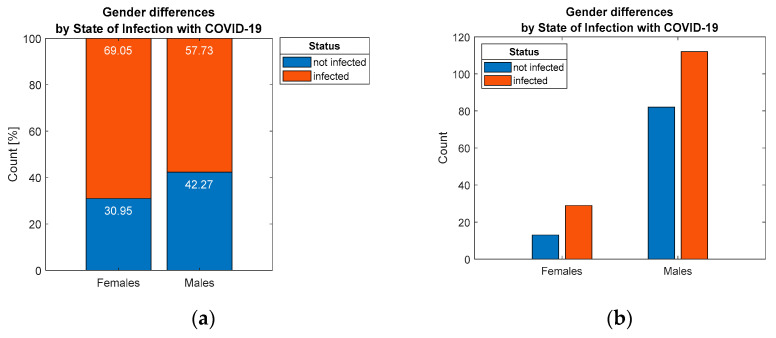
Distribution of respondents by: (**a**) percentage and (**b**) count, considering differentiation by gender.

In addition, the hypothesisH3 was not validated, and there is no statistically significant association (*p* = 0.175) related to the prevalence of infection among residents due belonging to a specific gender group, which is also illustrated by the proportions in Figure 5.

**Figure 6:**
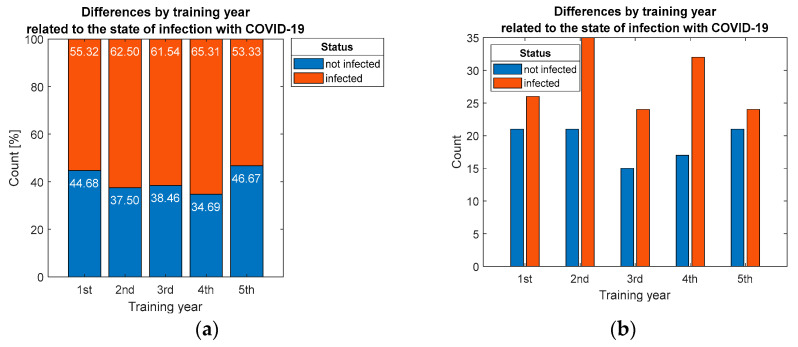
Distribution of respondents by: (**a**) percentage and (**b**) count considering differentiation by training year.

Correlation between the year of study in residency with the risk of infection, evaluated in hypothesis H4, demonstrated that no statistically significant association (*p* = 0.733) could be established (Figure 6).

**Figure 7:**
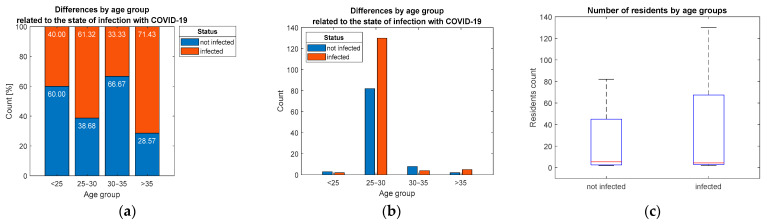
Distribution of respondents by: (**a**) percentage, (**b**,**c**) count considering differentiation by age group.

The test for the statistically significant association between age group and infection prevalence among residents did not confirm hypothesis H5, and there is no statistically significant association (*p* = 0.178), as is also illustrated by proportions in Figure 7.

**Figure 8:**
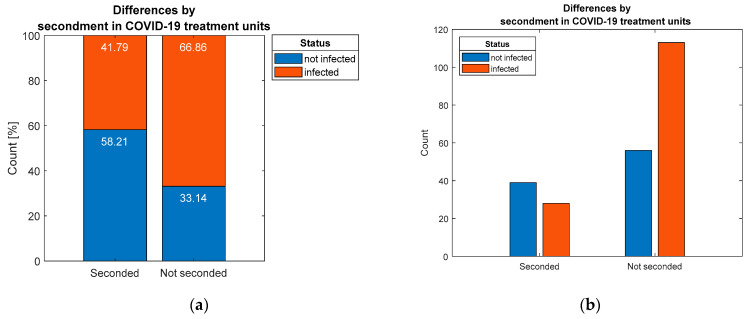
Distribution of respondents by: (**a**) percentage and (**b**) count, considering differentiation by secondment in COVID-19 units.

Furthermore, regarding the influence of secondment in COVID-19units on the increased risk of infection, formulated in hypothesis H6, this was validated by test because a statistically significant association (*p* = 0.0003) was established (Figure 8).

**Figure 9:**
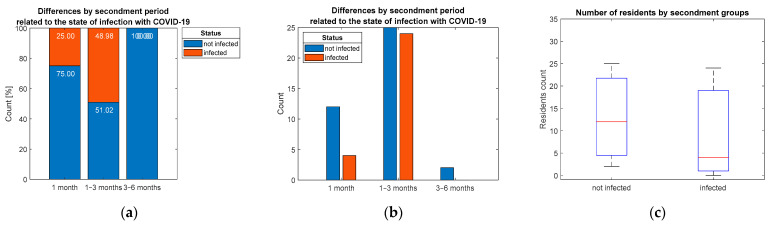
Distribution of respondents by: (**a**) percentage, (**b**,**c**) count considering differentiation by secondment period groups.

The statistical evaluation of hypothesis H7 demonstrates no significant association (*p* = 0.114) between the secondment period, and the prevalence of COVID-19 infections of the interviewed residents (Figure 9).

### 3.5. Assessment of Well-Being

A relatively large number of residents who participated in this study have a low level of well-being, which is revealed by the general WHO well-being index of residents, with values of 13.8 ± 5.7. Table 4 shows the proportion of residents who have a personal preference for a new residency specialization. Along with this, there are residents who would choose a non-medical career, who have an average well-being index of 12.3 ± 5.7. By comparison, residents who preferred to continue their medical career have a higher index value (14.2 ± 5.6, *p* = 0.024). The WHO average well-being index of residents with a preference for a new medical specialization was 12.5 ± 5.4, which is also a statistically significant difference if it is compared with the index value of residents who follow their chosen specialization (14.2 ± 5.7, *p* = 0.015). The recorded values of well-being levels are explained due to the confirmation of hypotheses 1 and 6, whereby the risk of infection is associated with the treatment of patients with COVID-19, and secondment in COVID-19 units.

### 3.6. Other Issues Considered Relevant

Residents’ responses highlighted a number of factors, and for this reason, the comments obtained in the open-ended question were reported in themes, together with the results of the quantitative survey to which they were most aligned. Table 5 displays summarized qualitative comments by theme and frequency.

Most participants in this research have expressed concerns about the risk of infection during work and quarantine.

The most frequent proposals in the final open-ended question of the survey suggested more transparency/predictability in terms of decisions made on the medical curriculum.

Many residents considered that general learning through web platforms is easier than before, and suggested that this form of training should be maintained and continued, possibly with the development of videos by managers of the training programs.

## 4. Discussion

Through this research, we aimed to evaluate the repercussion of the coronavirus disease on the professional training, career option, and mental condition of physicians residing in the specialty of orthopedics-traumatology in Romania.

A significant number of residents chose to volunteer in COVID-19 units (28.39%). This relocation of residents, and the reduction of elective interventions, were not in the interest of orthopedics residents’ training. However, Civantos et al. [22] point out that the cancellation of elective procedures, and the measures to limit internal staff, can have the effect of increasing the length of time spent on duty, mitigating some of the negative effects of the pandemic on the health of sanitary workers.

In the process of training as a specialist, residents are faced with challenging elements through which they must adapt to new environments, which is where they develop their professional identity [23]. Clinical education experiences are determining factors in choosing a medical career [24]. Practicing medicine as a profession may be uncertain for some residents, and a lack of tolerance for uncertainty may be a relevant predictor of psychological distress for them [25].

The first important findings of this study were that orthopedic residents had difficulty performing routine work during the COVID-19 pandemic, such as examining patients, obtaining information from other departments, procuring, and preparing implants for surgery.

It was found in this study that during the pandemic a large proportion of clinical activities (Figure 1) became more difficult. This finding can be explained by the redistribution of a large number of health workers from their regular positions to COVID-19 patient care units. However, the ability to manage the volume of patients in orthopedic wards was assessed as easier, due to the diminished number of cases duringthe pandemic. A significant number of residents had difficulty obtaining personal protective equipment at their workplaces, although the highly contagious nature of the infection was known. This has led to increased anxiety about the risk of COVID-19 contamination, which has been exacerbated by frequent changes in COVID-19 prevention and management strategies.

Our findings are consistent with the research conducted by Emre et al. [26] according to which, working in a ward serving patients with COVID-19, contact with positive patients and lack of personal protective equipment were risk factors for post-traumatic stress disorder in resident physicians.

The participants in our study completed their professional training by master’s or doctorate (Table 2), being involved in scientific research. This activity had been hampered by the difficulties encountered by doctoral students in conducting prospective/retrospective research (Figure 2), mainly due to a sharp decrease in the number of patients visiting hospitals with orthopedic problems.

The study by An et al. [27] points out that the education of orthopedic residents has continued to be successful with the rapid adoption of digital technologies, but the consequences of lost surgical experience remain unclear. The experiences of elective interventions, highly appreciated in the process of making career decisions in medical training, have disrupted the accumulation of specialized practical knowledge. In our study, the PhD students, who are also university assistants and conductpractical activities, reported a decrease in the level of interaction with students, due to the development of activities in virtual format, without the possibility of medical practice in the hospital ward.

Our study found that 45.34% of residents who participated in the study had higher than usual stress levels during the pandemic (Table 3). We appreciate that this condition is an effect of the challenges encountered during the pandemic, which was an unprecedented event.

The report on the state of higher education in Romania for the academic year 2019/2020 shows that the lowest losses (through repetition, unfinished situations, or dropout along the way) are recorded by study groups in Health and Social Work [28]. As shown in Table 4, our study found that 14.83% of participants reconsidered their residency choice, and 1.27% of residents reconsidered medicine as a career during this pandemic, which is in line with the trends highlighted in the national report on higher education.

Aljehani et al. [29] show that orthopedic residents have been affected by a considerable amount of stress, and hospitals need to make recommendations in order to guide them. Career orientation is affected by gender discrimination [30], limited participation in surgery [31], and personality differences in relation to the surgeons who must train them [32]. Along with these, perspective change in professional orientation may be due to the mental state of residents during the pandemic.

In our study, the well-being section of the WHO questionnaire showed that a considerable number of respondents had a WHO well-being index below 14, which suggests poor well-being, and requires psychological support [20]. Residents who reconsidered the choice of residency specialization, or who reconsidered medicine as a career, had significantly lower average WHO well-being rates, as the risk of infection is associated with the treatment of patients with COVID-19, and secondment in COVID-19 units.

In addition, our study also identified several factors that could explain this increase in stress during the pandemic (Figure 1); impaired activity; risk of COVID-19 infection; accumulation of practical surgical experience; and difficulties in obtaining information from other departments. Interestingly, residents were most affected by communication difficulties with other departments.

The study by Maunder et al. [33] shows that increasing interpersonal communication reduces the anxiety and stress that residents go through. Costa et al. [34] points out that the COVID-19 pandemic has adversely affected the health of resident physicians by increasing sleep disorders and mood swings. In order to reduce stress at work, policies need to be promoted to minimize burnout [35], while constantly monitoring them [36], to proactively identify residents who are at risk of psychological sequelae due to isolation [37].

The training of orthopedic residents has been profoundly affected by the pandemic. Most residents have been in favor of new learning methods by incorporating technology into their teaching methods, and with the pandemic, many have been able to quickly transition their teaching sessions to online formats. This can provide a solid basis for permanently incorporating distance learning into medicine.

Training videos for procedural learning with the intervention of hospital heads, residency training program managers, or medical opinion leaders answering residents’ questions, may be training routes for future orthopedic physicians.

In our study, we found that this crisis was an opportune moment for a deep debate to be induced, through which decision makers of medical programs can substantially change the way residents’ training is conducted. It is also essential for medical students to develop positive coping skills in order to benefit from these changes during their training, and in their future career, which is inherently stressful, as shown in the study by Zvauya et al. [38].

A limitation of our study is that although all residency training centers in Romania participated, the registration of residents in the survey was optional, without a rigorous statistical selection, which would have eliminated any errors.

The results of the study are deduced based on the answers collected from orthopedic residents in Romania, which cannot be generalized globally, because other national health systems have different infrastructures and organization systems, as well as training programs for residents [39].

Moreover, some elements of the survey were designed to be as specific as possible with regards to the reality of Romanian medical residents.

As such, we may have introduced errors in measuring results through the wording of the questions, and the means of collecting the answers. In general, the results of this survey should be seen as a qualitative and descriptive assessment of the overall impact of the pandemic on Romanian residents in the field of orthopedics, rather than an analytical assessment.

Furthermore, the limits of the study are due to the rapid transformations that are registered in health systems through the application of new strategies, and of the continuous changes in medical training, making it difficult to quantify the effectiveness of current strategies compared to previous routine protocols.

Future research directions may include other residency medical specializations, other national contexts, or other related qualitative issues [40].

## 5. Conclusions

The COVID-19 pandemic forced orthopedic-traumatology residents to adapt to a unique situation, in order to achieve their training objectives in their medical specialty. The pandemic has led to considerable stress among residents, which training centers should consider.

This study can serve as a reference for training center leaders when making important decisions on behalf of their residents. The results of our research may help residency training centers to develop robust programs that can survive this pandemic [41].

Although there is currently a global recession of the pandemic, its evolution is still unpredictable. However, some substantial changes are needed in residency training programs around the world, and the results of the research could shed light on this decision-making path. Emphasis should be placed on electronic educational portfolios, simulation of surgical procedures, and distance learning, all of which have a high potential for health safety and moral support for residents. At the same time, by adopting these changes and the widespread use of telemedicine, this can lead to better patient care.

The e-learning process may prove useful and may be incorporated into the long-term residency training program.

For all of these strategies to succeed, residents need to feel that they are concerned about their situation and are protected by the measures that apply. Collecting and processing feedback from residents, as well as taking action, will help the orthopedic community cope with the challenges of this pandemic.

## Figures and Tables

**Table 1:** Characteristics of the residents who participated in the study.

Characteristic	Number	Percent
*Residency Training Center*		
Arad	11	4.66
Brasov	14	5.93
Bucharest	32	13.56
Cluj-Napoca	23	9.75
Constanta	11	4.66
Craiova	12	5.09
Galati	18	7.63
Iasi	28	11.86
Oradea	16	6.78
Sibiu	19	8.05
Targu Mures	25	10.59
Timisoara	27	11.44
*Year of preparation for residency*		
1	47	19.92
2	56	23.73
3	39	16.52
4	49	20.76
5	45	19.07
*Age (years)*		
<25	5	2.12
25–30	212	89.83
30–35	12	5.08
>35	7	2.97
*Sex*		
Male	194	82.20
Female	42	17.80
*COVID positive patients were treated*		
Yes	170	72.03
No	66	27.97
*They were seconded to the COVID sections*		
Yes	67	28.39
1 month	16	6.78
1–3 months	49	20.76
3–6 months	2	0.85
No	169	71.61
*Tested positive*		
Yes	141	59.75
No	95	40.25
*Quarantine*		
Yes	141	59.75
No	95	40.25

**Table 2:** Characteristics of the residents who managed to improve their training through academic activity.

Characteristic	Number	Percent
Enrolled in master’s studies	47	19.92
Enrolled in a doctorate	23	9.75
Involved in scientific research activities	34	14.41
Involved in the professional training of students	29	3.4

**Table 3:** Residents’ level of stress during the pandemic.

Characteristic	Number	Percent
The level of stress higher than usual	107	45.34
The level of stress about the same as usual	117	49.58
The level of stress lower than usual	12	5.08

**Table 4:** Personal preference for a new residency specialization.

Characteristic	Number	Percent
Intensive care medicine	7	2.97
Emergency medicine	3	1.27
Microbiology	2	0.85
Specialties with a lower risk of contamination	14	5.93
Public health	4	1.69
Other	3	1.27
NA	2	0.84

**Table 5:** Synthesis of qualitative findings by theme for the open-ended question.

Construct	Number of Residents	Themes
Concerns	146	Risk of infection during work
	123	Quarantine
	68	A new wave of epidemics
Proposals	78	More transparency for decisions made on the medical curriculum
	46	More predictability about decision on the medical curriculum
	29	Guidelines for blended learning
Improvements in the training programme	57	General learning through web platforms
	39	Development of videos for training
	24	Simulation laboratory

## Data Availability

The data presented in this study are available on request from the corresponding author.
